# 2-(4-Fluoro­phen­yl)-1-(4-meth­oxy­phen­yl)-4,5-dimethyl-1*H*-imidazole

**DOI:** 10.1107/S1600536810024104

**Published:** 2010-06-26

**Authors:** P. Gayathri, J. Jayabharathi, K. Saravanan, A. Thiruvalluvar, R.J. Butcher

**Affiliations:** a PG Research Department of Physics, Rajah Serfoji Government College (Autonomous), Thanjavur 613 005, Tamilnadu, India; b Department of Chemistry, Annamalai University, Annamalai Nagar 608 002, Tamilnadu, India; c Department of Chemistry, Howard University, 525 College Street NW, Washington, DC 20059, USA

## Abstract

In the title compound, C_18_H_17_FN_2_O, the imidazole ring makes dihedral angles of 76.46 (7) and 40.68 (7)° with the meth­oxy­phenyl and fluoro­phenyl rings, respectively. The dihedral angle between the two benzene rings is 71.25 (6)°.

## Related literature

For the optical properties of heterocyclic imidazole derivatives, see: Santos *et al.* (2001 ▶); Huang *et al.* (2004 ▶). For their role in the preparation of functionalized materials, see: Kamidate *et al.* (1989 ▶). For their fluorescence and chemiluminescence properties, see: Ucucu *et al.* (2001 ▶). For their use in the construction of fluorescent chemisensors, see: Jayabharathi *et al.* (2009 ▶, 2010 ▶); Zhou & Fahrni (2004 ▶).
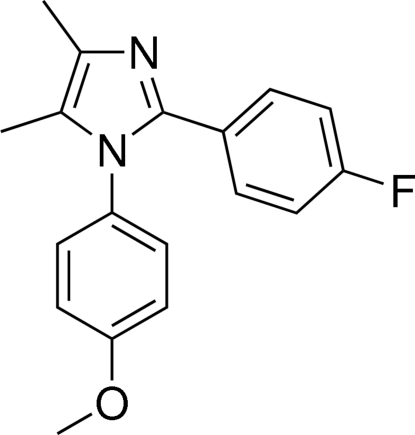


## Experimental

### 

#### Crystal data

C_18_H_17_FN_2_O
*M* *_r_* = 296.34
Monoclinic, 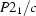

*a* = 8.5132 (1) Å
*b* = 9.5128 (2) Å
*c* = 19.2610 (3) Å
β = 96.798 (2)°
*V* = 1548.87 (4) Å^3^
*Z* = 4
Cu *K*α radiation
μ = 0.72 mm^−1^
*T* = 295 K
0.40 × 0.32 × 0.22 mm

#### Data collection

Oxford Diffraction Xcalibur Ruby Gemini diffractometer
Absorption correction: multi-scan (*CrysAlis PRO*; Oxford Diffraction, 2010 ▶) *T*_min_ = 0.835, *T*_max_ = 1.000
6517 measured reflections
3235 independent reflections
2744 reflections with *I* > 2σ(*I*)
*R*_int_ = 0.018

#### Refinement

*R*[*F* ^2^ > 2σ(*F* ^2^)] = 0.041
*wR*(*F* ^2^) = 0.130
*S* = 1.08
3235 reflections
200 parameters
H-atom parameters constrained
Δρ_max_ = 0.16 e Å^−3^
Δρ_min_ = −0.20 e Å^−3^

### 

Data collection: *CrysAlis PRO* (Oxford Diffraction, 2010 ▶); cell refinement: *CrysAlis PRO*; data reduction: *CrysAlis PRO*; program(s) used to solve structure: *SHELXS97* (Sheldrick, 2008 ▶); program(s) used to refine structure: *SHELXL97* (Sheldrick, 2008 ▶); molecular graphics: *ORTEP-3* (Farrugia, 1997 ▶); software used to prepare material for publication: *PLATON* (Spek, 2009 ▶).

## Supplementary Material

Crystal structure: contains datablocks global, I. DOI: 10.1107/S1600536810024104/wn2396sup1.cif

Structure factors: contains datablocks I. DOI: 10.1107/S1600536810024104/wn2396Isup2.hkl

Additional supplementary materials:  crystallographic information; 3D view; checkCIF report

## Figures and Tables

**Fig. 1.**
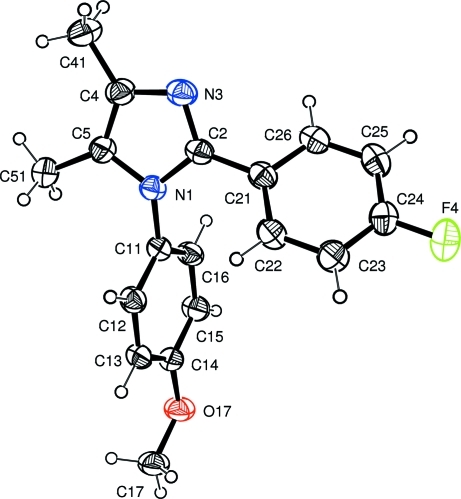
The molecular structure of the title compound, showing the atom-numbering scheme and displacement ellipsoids drawn at the 30% probability level. H atoms are shown as small spheres of arbitrary radius.

## supplementary crystallographic information

### Comment

Recently heterocyclic imidazole derivatives have attracted considerable attention because of their unique optical properties (Santos *et al.*, (2001) and Huang *et al.*, (2004)). These compounds play a very important role in chemistry as mediators for synthetic reactions, primarily for preparing functionalized materials (Kamidate *et al.*, (1989)). The imidazole nucleus forms the main structure of some well known components of human organisms, *e.g.*, the amino acid histidine, Vitamin B12, a component of DNA base structure, purines, histamine and biotin and is present in many natural or synthetic drug molecules, *e.g.*, azomycin, cimetidine and metronidazole. These also have significant analytical applications utilizing their fluorescence and chemiluminescence properties (Ucucu *et al.*, (2001)). An important property that makes imidazole derivatives more attractive as a chelator is the appreciable change in its fluorescence upon metal binding. Therefore, imidazole derivatives have been used to construct highly sensitive fluorescent chemisensors for sensing and imaging of metal ions. Its chelates in particular those with Ir^3+^ are major components for organic light-emitting diodes (Jayabharathi *et al.*, (2009)) and are promising candidates for fluorescent chemisensors (Zhou & Fahrni (2004)) for metal ions. In this paper we report the crystal and molecular structure of the title compound, a fluorescent chemisensor (Jayabharathi *et al.*, (2010)), synthesized in our laboratory.

In the title molecule (Fig. 1), C_18_H_17_FN_2_O, the imidazole ring is essentially planar [maximum deviation of 0.005 (1) Å for C2]. The imidazole ring makes dihedral angles of 76.46 (7)° and 40.68 (7)° with the methoxyphenyl (C11–C16) and fluorophenyl (C21–C26) rings, respectively. The dihedral angle between the two benzene rings is 71.25 (6)°. In the crystal structure no classical hydrogen bonds are observed.

### Experimental

To pure biacetyl (1.48 g, 15 mmol) in ethanol (10 ml), *p*-anisidine (1.84 g, 15 mmol) ammonium acetate (7.0 g, 15 mmol) and 4-fluorobenzaldehyde (1.8 g, 15 mmol) were added over a period of about 1 h, maintaining the temperature at 333 K. The reaction mixture was refluxed for 7 days and extracted with dichloromethane. The solid which separated was purified by column chromatography using hexane: ethyl acetate as the eluent. Yield: 1.77 g (40%).

### Refinement

H atoms were positioned geometrically and allowed to ride on their parent atoms, with C—H = 0.93 Å for Csp^2^ and 0.96 Å for Csp^3^; *U*_iso_(H) = k*U*_eq_(C), where k = 1.5 for methyl and 1.2 for all other H atoms.

### Crystal data

**Table:**

C_18_H_17_FN_2_O	*F*(000) = 624
*M**_r_* = 296.34	*D*_x_ = 1.271 Mg m^−^^3^
Monoclinic, *P*2_1_/*c*	Melting point: 385 K
Hall symbol: -P 2ybc	Cu *K*α radiation, λ = 1.54184 Å
*a* = 8.5132 (1) Å	Cell parameters from 4271 reflections
*b* = 9.5128 (2) Å	θ = 4.6–77.3°
*c* = 19.2610 (3) Å	µ = 0.72 mm^−^^1^
β = 96.798 (2)°	*T* = 295 K
*V* = 1548.87 (4) Å^3^	Prism, colourless
*Z* = 4	0.40 × 0.32 × 0.22 mm

### Data collection

**Table:**

Oxford Diffraction Xcalibur Ruby Gemini diffractometer	3235 independent reflections
Radiation source: Enhance (Cu) X-ray Source	2744 reflections with *I* > 2σ(*I*)
graphite	*R*_int_ = 0.018
Detector resolution: 10.5081 pixels mm^-1^	θ_max_ = 77.5°, θ_min_ = 4.6°
ω scans	*h* = −9→10
Absorption correction: multi-scan (*CrysAlis PRO*; Oxford Diffraction, 2010)	*k* = −12→9
*T*_min_ = 0.835, *T*_max_ = 1.000	*l* = −23→24
6517 measured reflections	

### Refinement

**Table:**

Refinement on *F*^2^	Secondary atom site location: difference Fourier map
Least-squares matrix: full	Hydrogen site location: inferred from neighbouring sites
*R*[*F*^2^ > 2σ(*F*^2^)] = 0.041	H-atom parameters constrained
*wR*(*F*^2^) = 0.130	*w* = 1/[σ^2^(*F*_o_^2^) + (0.076*P*)^2^ + 0.1231*P*] where *P* = (*F*_o_^2^ + 2*F*_c_^2^)/3
*S* = 1.08	(Δ/σ)_max_ = 0.001
3235 reflections	Δρ_max_ = 0.16 e Å^−^^3^
200 parameters	Δρ_min_ = −0.20 e Å^−^^3^
0 restraints	Extinction correction: *SHELXL97* (Sheldrick, 2008), Fc^*^=kFc[1+0.001xFc^2^λ^3^/sin(2θ)]^-1/4^
Primary atom site location: structure-invariant direct methods	Extinction coefficient: 0.0214 (14)

### Special details

**Table:**

Geometry. Bond distances, angles *etc*. have been calculated using the rounded fractional coordinates. All su's are estimated from the variances of the (full) variance-covariance matrix. The cell e.s.d.'s are taken into account in the estimation of distances, angles and torsion angles
Refinement. Refinement of *F*^2^ against ALL reflections. The weighted *R*-factor *wR* and goodness of fit *S* are based on *F*^2^, conventional *R*-factors *R* are based on *F*, with *F* set to zero for negative *F*^2^. The threshold expression of *F*^2^ > 2σ(*F*^2^) is used only for calculating *R*-factors(gt) *etc*. and is not relevant to the choice of reflections for refinement. *R*-factors based on *F*^2^ are statistically about twice as large as those based on *F*, and *R*- factors based on ALL data will be even larger.

### Fractional atomic coordinates and isotropic or equivalent isotropic displacement parameters (Å^2^)

**Table:**

	*x*	*y*	*z*	*U*_iso_*/*U*_eq_	
F4	0.28268 (12)	−0.00876 (12)	0.68530 (5)	0.0874 (4)	
O17	0.19515 (13)	−0.00279 (11)	0.26852 (6)	0.0708 (4)	
N1	0.26219 (12)	0.46448 (11)	0.44385 (5)	0.0517 (3)	
N3	0.22859 (14)	0.59440 (12)	0.53660 (6)	0.0604 (4)	
C2	0.24814 (14)	0.46597 (14)	0.51401 (6)	0.0530 (4)	
C4	0.22762 (16)	0.68002 (15)	0.47855 (7)	0.0623 (4)	
C5	0.24779 (16)	0.60275 (14)	0.42099 (7)	0.0581 (4)	
C11	0.25104 (14)	0.34332 (12)	0.39879 (6)	0.0480 (3)	
C12	0.38038 (14)	0.29589 (14)	0.36872 (6)	0.0531 (4)	
C13	0.36702 (15)	0.18029 (14)	0.32457 (6)	0.0540 (4)	
C14	0.22295 (15)	0.11173 (13)	0.31098 (6)	0.0529 (4)	
C15	0.09313 (16)	0.16030 (15)	0.34140 (7)	0.0609 (4)	
C16	0.10672 (14)	0.27585 (14)	0.38472 (7)	0.0561 (4)	
C17	0.3214 (2)	−0.05517 (17)	0.23377 (8)	0.0719 (5)	
C21	0.25773 (14)	0.33905 (14)	0.55796 (6)	0.0531 (4)	
C22	0.36774 (16)	0.23360 (16)	0.55099 (7)	0.0617 (4)	
C23	0.37767 (17)	0.11663 (17)	0.59375 (7)	0.0664 (5)	
C24	0.27481 (16)	0.10690 (16)	0.64337 (7)	0.0629 (4)	
C25	0.16576 (18)	0.20821 (18)	0.65268 (7)	0.0684 (5)	
C26	0.15801 (13)	0.32536 (12)	0.60982 (6)	0.0620 (4)	
C41	0.20446 (13)	0.83510 (12)	0.48384 (6)	0.0871 (7)	
C51	0.2531 (2)	0.64214 (17)	0.34669 (8)	0.0771 (6)	
H12	0.47707	0.34170	0.37811	0.0636*	
H13	0.45430	0.14889	0.30417	0.0647*	
H15	−0.00361	0.11441	0.33243	0.0731*	
H16	0.01913	0.30851	0.40450	0.0674*	
H17A	0.40446	−0.08890	0.26772	0.1079*	
H17B	0.28354	−0.13078	0.20326	0.1079*	
H17C	0.36133	0.01881	0.20685	0.1079*	
H22	0.43594	0.24196	0.51689	0.0740*	
H23	0.45182	0.04651	0.58909	0.0797*	
H25	0.09829	0.19875	0.68700	0.0820*	
H26	0.08516	0.39590	0.61576	0.0744*	
H41A	0.09876	0.85400	0.49396	0.1306*	
H41B	0.27883	0.87228	0.52062	0.1306*	
H41C	0.22090	0.87872	0.44034	0.1306*	
H51A	0.22550	0.73950	0.34023	0.1157*	
H51B	0.35792	0.62701	0.33449	0.1157*	
H51C	0.17938	0.58537	0.31733	0.1157*	

### Atomic displacement parameters (Å^2^)

**Table:**

	*U*^11^	*U*^22^	*U*^33^	*U*^12^	*U*^13^	*U*^23^
F4	0.0794 (6)	0.0928 (7)	0.0893 (6)	−0.0048 (5)	0.0075 (5)	0.0315 (5)
O17	0.0808 (7)	0.0606 (6)	0.0735 (6)	−0.0040 (5)	0.0191 (5)	−0.0211 (5)
N1	0.0556 (5)	0.0500 (5)	0.0508 (5)	−0.0028 (4)	0.0114 (4)	−0.0055 (4)
N3	0.0624 (6)	0.0605 (7)	0.0591 (6)	−0.0047 (5)	0.0109 (5)	−0.0151 (5)
C2	0.0516 (6)	0.0578 (7)	0.0502 (6)	−0.0020 (5)	0.0080 (5)	−0.0098 (5)
C4	0.0626 (8)	0.0534 (7)	0.0723 (8)	−0.0063 (5)	0.0143 (6)	−0.0103 (6)
C5	0.0611 (7)	0.0511 (7)	0.0641 (7)	−0.0044 (5)	0.0152 (5)	−0.0028 (5)
C11	0.0525 (6)	0.0479 (6)	0.0448 (5)	0.0004 (5)	0.0103 (4)	−0.0021 (4)
C12	0.0486 (6)	0.0571 (7)	0.0546 (6)	−0.0029 (5)	0.0109 (5)	−0.0009 (5)
C13	0.0558 (7)	0.0566 (7)	0.0518 (6)	0.0074 (5)	0.0161 (5)	0.0000 (5)
C14	0.0644 (7)	0.0479 (6)	0.0475 (6)	0.0019 (5)	0.0114 (5)	−0.0021 (5)
C15	0.0546 (7)	0.0617 (8)	0.0681 (8)	−0.0097 (5)	0.0144 (6)	−0.0120 (6)
C16	0.0505 (6)	0.0593 (7)	0.0614 (7)	−0.0011 (5)	0.0183 (5)	−0.0083 (6)
C17	0.0900 (10)	0.0660 (8)	0.0607 (8)	0.0139 (7)	0.0130 (7)	−0.0143 (7)
C21	0.0521 (6)	0.0609 (7)	0.0457 (6)	−0.0053 (5)	0.0029 (4)	−0.0085 (5)
C22	0.0580 (7)	0.0754 (9)	0.0526 (7)	0.0050 (6)	0.0104 (5)	0.0022 (6)
C23	0.0619 (7)	0.0734 (9)	0.0630 (8)	0.0089 (7)	0.0035 (6)	0.0035 (7)
C24	0.0598 (7)	0.0707 (8)	0.0560 (7)	−0.0085 (6)	−0.0019 (5)	0.0060 (6)
C25	0.0640 (8)	0.0852 (10)	0.0574 (7)	−0.0100 (7)	0.0136 (6)	0.0001 (7)
C26	0.0602 (7)	0.0692 (8)	0.0579 (7)	−0.0010 (6)	0.0125 (5)	−0.0090 (6)
C41	0.1054 (13)	0.0557 (9)	0.1035 (13)	−0.0034 (8)	0.0256 (10)	−0.0168 (8)
C51	0.1017 (12)	0.0617 (9)	0.0721 (9)	0.0013 (8)	0.0276 (8)	0.0082 (7)

### Geometric parameters (Å, °)

**Table:**

F4—C24	1.3618 (18)	C23—C24	1.374 (2)
O17—C14	1.3660 (16)	C24—C25	1.365 (2)
O17—C17	1.422 (2)	C25—C26	1.384 (2)
N1—C2	1.3707 (15)	C12—H12	0.9300
N1—C5	1.3879 (17)	C13—H13	0.9300
N1—C11	1.4392 (15)	C15—H15	0.9300
N3—C2	1.3141 (17)	C16—H16	0.9300
N3—C4	1.3825 (18)	C17—H17A	0.9600
C2—C21	1.4713 (18)	C17—H17B	0.9600
C4—C5	1.3579 (19)	C17—H17C	0.9600
C4—C41	1.4934 (18)	C22—H22	0.9300
C5—C51	1.485 (2)	C23—H23	0.9300
C11—C12	1.3793 (17)	C25—H25	0.9300
C11—C16	1.3842 (17)	C26—H26	0.9300
C12—C13	1.3865 (18)	C41—H41A	0.9600
C13—C14	1.3864 (18)	C41—H41B	0.9600
C14—C15	1.3898 (19)	C41—H41C	0.9600
C15—C16	1.3766 (19)	C51—H51A	0.9600
C21—C22	1.3897 (19)	C51—H51B	0.9600
C21—C26	1.3915 (16)	C51—H51C	0.9600
C22—C23	1.381 (2)		
			
F4···H13^i^	2.5900	C26···H17C^vii^	2.8100
F4···H15^ii^	2.5600	C41···H51A	2.9400
F4···H17A^i^	2.8600	C51···H41C	2.9200
O17···H51A^iii^	2.8100	C51···H26^v^	3.0700
N1···H22	2.8600	H12···C17^ix^	3.0700
N3···H26	2.8000	H12···N3^iv^	2.8900
N3···H12^iv^	2.8900	H13···C17	2.5500
N3···H16^v^	2.6700	H13···H17A	2.3900
C4···C26^v^	3.5149 (18)	H13···H17C	2.3100
C5···C26^v^	3.5027 (18)	H13···F4^i^	2.5900
C11···C22	3.1592 (18)	H15···F4^ii^	2.5600
C12···C51	3.478 (2)	H16···C2	3.0900
C12···C22	3.5745 (18)	H16···N3^v^	2.6700
C14···C25^vi^	3.4808 (19)	H17A···C13	2.8200
C16···C21	3.4832 (18)	H17A···H13	2.3900
C17···C26^vi^	3.410 (2)	H17A···F4^i^	2.8600
C21···C16	3.4832 (18)	H17A···C23^i^	3.0800
C22···C11	3.1592 (18)	H17A···C24^i^	3.0500
C22···C12	3.5745 (18)	H17C···C13	2.7300
C25···C14^vii^	3.4808 (19)	H17C···H13	2.3100
C26···C17^vii^	3.410 (2)	H17C···C26^vi^	2.8100
C26···C4^v^	3.5149 (18)	H22···N1	2.8600
C26···C5^v^	3.5027 (18)	H22···C11	2.7800
C51···C12	3.478 (2)	H22···C12	2.8800
C2···H16	3.0900	H22···C4^iv^	2.9500
C4···H22^iv^	2.9500	H23···H41B^iii^	2.4900
C5···H26^v^	2.8400	H23···C13^i^	3.0300
C11···H22	2.7800	H25···C14^vii^	3.0800
C11···H51C	2.8100	H26···N3	2.8000
C12···H22	2.8800	H26···C5^v^	2.8400
C13···H17A	2.8200	H26···C51^v^	3.0700
C13···H23^i^	3.0300	H41B···C23^x^	2.8000
C13···H17C	2.7300	H41B···H23^x^	2.4900
C14···H25^vi^	3.0800	H41C···C51	2.9200
C17···H12^viii^	3.0700	H41C···H51A	2.3400
C17···H13	2.5500	H51A···O17^x^	2.8100
C17···H51A^iii^	3.0100	H51A···C17^x^	3.0100
C23···H17A^i^	3.0800	H51A···C41	2.9400
C23···H41B^iii^	2.8000	H51A···H41C	2.3400
C24···H17A^i^	3.0500	H51C···C11	2.8100
			
C14—O17—C17	118.31 (12)	C11—C12—H12	120.00
C2—N1—C5	106.75 (10)	C13—C12—H12	120.00
C2—N1—C11	126.56 (10)	C12—C13—H13	120.00
C5—N1—C11	124.79 (10)	C14—C13—H13	120.00
C2—N3—C4	105.59 (11)	C14—C15—H15	120.00
N1—C2—N3	111.43 (11)	C16—C15—H15	120.00
N1—C2—C21	123.60 (11)	C11—C16—H16	120.00
N3—C2—C21	124.96 (11)	C15—C16—H16	120.00
N3—C4—C5	110.62 (12)	O17—C17—H17A	109.00
N3—C4—C41	121.00 (11)	O17—C17—H17B	109.00
C5—C4—C41	128.38 (12)	O17—C17—H17C	109.00
N1—C5—C4	105.60 (11)	H17A—C17—H17B	109.00
N1—C5—C51	122.22 (12)	H17A—C17—H17C	109.00
C4—C5—C51	132.17 (13)	H17B—C17—H17C	109.00
N1—C11—C12	121.08 (11)	C21—C22—H22	119.00
N1—C11—C16	118.89 (11)	C23—C22—H22	119.00
C12—C11—C16	120.02 (11)	C22—C23—H23	121.00
C11—C12—C13	120.36 (11)	C24—C23—H23	121.00
C12—C13—C14	119.70 (11)	C24—C25—H25	121.00
O17—C14—C13	125.09 (12)	C26—C25—H25	121.00
O17—C14—C15	115.31 (12)	C21—C26—H26	120.00
C13—C14—C15	119.60 (12)	C25—C26—H26	120.00
C14—C15—C16	120.47 (12)	C4—C41—H41A	109.00
C11—C16—C15	119.86 (12)	C4—C41—H41B	109.00
C2—C21—C22	121.93 (11)	C4—C41—H41C	109.00
C2—C21—C26	119.53 (11)	H41A—C41—H41B	109.00
C22—C21—C26	118.50 (12)	H41A—C41—H41C	109.00
C21—C22—C23	121.22 (13)	H41B—C41—H41C	109.00
C22—C23—C24	118.05 (14)	C5—C51—H51A	109.00
F4—C24—C23	118.45 (13)	C5—C51—H51B	109.00
F4—C24—C25	118.63 (12)	C5—C51—H51C	109.00
C23—C24—C25	122.92 (14)	H51A—C51—H51B	109.00
C24—C25—C26	118.39 (13)	H51A—C51—H51C	109.00
C21—C26—C25	120.90 (12)	H51B—C51—H51C	109.00
			
C17—O17—C14—C13	−1.62 (19)	N3—C4—C5—C51	178.88 (15)
C17—O17—C14—C15	177.89 (12)	C41—C4—C5—N1	−179.32 (12)
C5—N1—C2—N3	−0.96 (14)	C41—C4—C5—C51	−0.3 (3)
C5—N1—C2—C21	−179.65 (11)	N1—C11—C12—C13	−179.00 (11)
C11—N1—C2—N3	−165.76 (11)	C16—C11—C12—C13	−0.29 (18)
C11—N1—C2—C21	15.55 (19)	N1—C11—C16—C15	179.58 (11)
C2—N1—C5—C4	0.64 (14)	C12—C11—C16—C15	0.84 (19)
C2—N1—C5—C51	−178.50 (13)	C11—C12—C13—C14	−0.36 (18)
C11—N1—C5—C4	165.78 (11)	C12—C13—C14—O17	179.95 (13)
C11—N1—C5—C51	−13.4 (2)	C12—C13—C14—C15	0.46 (18)
C2—N1—C11—C12	−113.75 (14)	O17—C14—C15—C16	−179.44 (12)
C2—N1—C11—C16	67.52 (16)	C13—C14—C15—C16	0.10 (19)
C5—N1—C11—C12	84.05 (16)	C14—C15—C16—C11	−0.8 (2)
C5—N1—C11—C16	−94.68 (15)	C2—C21—C22—C23	178.47 (12)
C4—N3—C2—N1	0.86 (14)	C26—C21—C22—C23	0.7 (2)
C4—N3—C2—C21	179.52 (12)	C2—C21—C26—C25	−179.05 (12)
C2—N3—C4—C5	−0.43 (16)	C22—C21—C26—C25	−1.26 (18)
C2—N3—C4—C41	178.82 (12)	C21—C22—C23—C24	0.3 (2)
N1—C2—C21—C22	40.96 (18)	C22—C23—C24—F4	179.35 (13)
N1—C2—C21—C26	−141.33 (12)	C22—C23—C24—C25	−1.0 (2)
N3—C2—C21—C22	−137.56 (14)	F4—C24—C25—C26	−179.85 (12)
N3—C2—C21—C26	40.16 (18)	C23—C24—C25—C26	0.5 (2)
N3—C4—C5—N1	−0.14 (15)	C24—C25—C26—C21	0.7 (2)

Symmetry codes: (i) −*x*+1, −*y*, −*z*+1; (ii) −*x*, −*y*, −*z*+1; (iii) *x*, *y*−1, *z*; (iv) −*x*+1, −*y*+1, −*z*+1; (v) −*x*, −*y*+1, −*z*+1; (vi) *x*, −*y*+1/2, *z*−1/2; (vii) *x*, −*y*+1/2, *z*+1/2; (viii) −*x*+1, *y*−1/2, −*z*+1/2; (ix) −*x*+1, *y*+1/2, −*z*+1/2; (x) *x*, *y*+1, *z*.

## References

[bb1] Farrugia, L. J. (1997). *J. Appl. Cryst.* **30**, 565.

[bb2] Huang, W. S., Lin, J. T., Chien, C. H., Tao, Y. T., Sun, S. S. & Wen, Y. S. (2004). *Chem. Mater.* **16**, 2480–2488.

[bb3] Jayabharathi, J., Thanikachalam, V. & Saravanan, K. (2009). *J. Photochem. Photobiol. A*, **208**, 13–20.

[bb4] Jayabharathi, J., Thanikachalam, V., Saravanan, K. & Srinivasan, N. (2010). *J. Fluorescence.* Accepted. 10.1007/s10895-010-0747-5 21046440

[bb5] Kamidate, T., Yamaguchi, K., Segawa, T. & Watanabe, H. (1989). *Anal. Sci.* **5**, 429–433.

[bb6] Oxford Diffraction (2010). *CrysAlis PRO* Oxford Diffraction Ltd, Yarnton, England.

[bb7] Santos, J., Mintz, E. A., Zehnder, O., Bosshard, C., Bu, X. R. & Günter, P. (2001). *Tetrahedron Lett.* **42**, 805–808.

[bb8] Sheldrick, G. M. (2008). *Acta Cryst.* A**64**, 112–122. 10.1107/S0108767307043930 18156677

[bb9] Spek, A. L. (2009). *Acta Cryst.* D**65**, 148–155. 10.1107/S090744490804362X PMC2631630 19171970

[bb10] Ucucu, U., Karaburun, N. G. & Isikdag, I. (2001). * Farmaco*, **56**, 285–290. 10.1016/s0014-827x(01)01076-x 11421256

[bb11] Zhou, Z. & Fahrni, C. J. (2004). *J. Am. Chem. Soc.* **126**, 8862–8863. 10.1021/ja049684r 15264794

